# Detection of Axitinib Using Multiwalled Carbon Nanotube-Fe_2_O_3_/Chitosan Nanocomposite-Based Electrochemical Sensor and Modeling with Density Functional Theory

**DOI:** 10.1021/acsomega.2c04244

**Published:** 2022-09-16

**Authors:** Ahmet Cetinkaya, S. Irem Kaya, Pelin Şenel, Nejla Cini, Esen B. Atici, Sibel A. Ozkan, Mine Yurtsever, Ayşegül Gölcü

**Affiliations:** † Department of Analytical Chemistry, Faculty of Pharmacy, Ankara University, 06560 Ankara, Turkiye; ‡ Department of Analytical Chemistry, Gulhane Faculty of Pharmacy, University of Health Sciences, 06010 Ankara, Turkiye; § Chemistry Department, Science and Letters Faculty, Istanbul Technical University, Maslak, 34469 Istanbul, Turkiye; ∥ Research & Development Center, DEVA Holding A.S., 59520 Tekirdağ, Turkiye

## Abstract

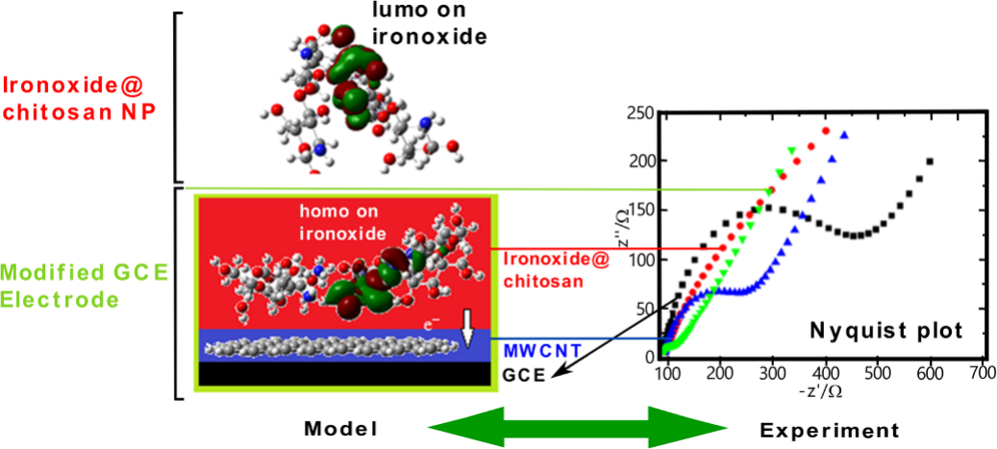

In this study, axitinib (AXI), a potent and selective inhibitor of vascular endothelial growth factor receptor (VEGFR) tyrosine kinase and used as a second-generation targeted drug, was investigated electrochemically under optimized conditions using multiwalled carbon nanotubes/iron(III) oxide nanoparticle–chitosan nanocomposite (MWCNT/Fe_2_O_3_@chitosan NC) modified on the glassy carbon electrode (GCE) surface. Characterization of the modified electrode was performed using scanning electron microscopy (SEM) and electrochemical impedance spectroscopy (EIS). The adsorptive stripping differential pulse voltammetric (AdSDPV) technique was used for the sensitive, rapid, and precise detection of AXI. The current peak obtained with the MWCNT/Fe_2_O_3_@chitosan NC modified electrode was 23 times higher compared to the bare electrode. The developed modified electrode showed excellent electrocatalytic activity in AXI oxidation. Under optimized conditions, the effect of supporting electrolyte and pH was investigated, and 0.1 M H_2_SO_4_ was chosen as the electrolyte with the highest peak current for the target analyte. In the concentration range of MWCNT/Fe_2_O_3_@chitosan NC/GCE, 6 × 10^–9^ and 1 × 10^–6^ M, the limit of detection (LOD) and limit of quantification (LOQ) values were calculated to be 0.904 and 0.0301 pM, respectively. Tablet and serum samples were used for the applicability of the developed sensor, relative standard deviation (RSD) values for all samples were below 2%, and the recovery results were 99.23 and 101.84%, respectively. The MWCNT/Fe_2_O_3_@chitosan NC/GCE designed to determine AXI demonstrated the applicability, selectivity, precision, and accuracy of the sensor. The mechanism of electron transfer from the modified GCE surface to the analyte solution is studied via modeling the modified GCE surface by the density functional theory (DFT) method at B3LYP/6-311+g(d,p) and M062X/6-31g(d,p) levels. We observed that the iron oxide nanoparticles play an important role in channeling electron flow from the analyte solution to the MWCNT-coated GCE electrode surface. Adsorption of the nanocomposite material onto the GCE surface occurs via strong electrostatic interactions, including ionic and hydrogen bond formations. During the adsorption-controlled oxidation process of the axitinib, the electrons are transferred via the highest occupied molecular orbital (HOMO) localized on the iron oxide moiety to the lowest unoccupied molecular orbital (LUMO) of the MWCNT/GCE surface.

## Introduction

Despite the developments in the pharmaceutical industry and medicine, cancer-related deaths are still in the first place worldwide. For this reason, research on the causes of carcinogenesis has gained importance. Vascular endothelial growth factor receptor (VEGFR) tyrosine kinase-related pathways are highly associated with tumor metastasis and angiogenesis.^1^ Tyrosine kinase inhibitors such as sorafenib, imatinib, sunitinib, dasatinib, etc., are approved and used clinically to treat different types of cancers. Axitinib (AXI) has a highly potent and selective inhibitory activity on VEGFR-1, VEGFR-2, and VEGFR-3 tyrosine kinase. Studies continue on the use of AXI, which stands out for its higher inhibitory activity than other tyrosine kinase inhibitors, in different types of cancer (such as nonsmall cell lung cancer and thyroid cancer).^2−4^ When the literature is evaluated, it can be seen that various analytical techniques are employed for the AXI determination, such as micellar liquid chromatography,^5^ liquid chromatography-tandem mass spectrometry (LC-MS/MS),^6,7^ ion mobility spectrometry, direct analysis in real-time (DART) mass spectrometry,^8^ and fluorescence spectroscopy.^9^ Even though these techniques enable selective, sensitive, and accurate analysis, they suffer from high costs, complex procedures, and long analysis times. As an alternative to these techniques, electrochemical sensors offer high sensitivity, low cost, environmental- and user-friendliness, ease of use, portability, and short analysis time.^10,11^ In addition, the fabrication of environmentally friendly sensing systems with low emission and high selectivity at low cost is very important. Taken together, further developments of sensing materials with improved advantageous features by employing innovative nanotechnology strategies, which lead to the design of ultrasmall device mechanisms, is essential.^12−15^ In fact, the improvement of the sensing properties in modern technologies, as well as re-evaluation of their usability, has been the main challenging task in the current research studies.

There are different strategies to improve the performance of electrochemical sensors, and modification with nanomaterials is the most effective and widely used strategy. In this concept, nanoarchitectonics^16,17^ have emerged for designing functional materials in nanoscale units with high-level structural regulations and attracted interest in the last decade for sensing and detection purposes in the analytical method development and related instruments.^18,19^ Considering the sensors and construction of respective devices as the active targets of nanoarchitectonics, interactions and multiple processes are combined to provide more advanced results and hierarchical structures for better sensing and molecular recognition purposes.^19^

The enhancement of the interfacial area, providing a facile contact between the sensing target molecules and sensor device material, is one of the effective ways to increase the sensitivity of sensors.^16−24^ Due to the unique properties of nanomaterials, such as great electrocatalytic activity and high conductivity, nanomaterial-based electrochemical sensors can provide highly sensitive, efficient, accurate, and precise analysis.^11,20,25^ In that respect, nanoporous structures with highly enhanced molecular sensing capability at surfaces obtained by molecular self-assembly and template synthesis are good examples of nanoarchitectonics application strategies.^16−19,23^ It has been revealed that nanoparticles and nanoporous materials are advantageous for improved sensor performance owing to their high surface area.^19,23,26^ There is only one study in the literature that uses an unmodified glassy carbon electrode (GCE) and boron-doped diamond electrode (BDDE) for AXI determination.^2^ This work evaluated the electrochemical behavior of AXI on bare electrodes in detail. On the other hand, our study aims to develop a nanomaterial-based sensor platform to reach a low limit of detection (LOD) value by increasing the sensitivity and creating a sensor with higher performance.

In this study, an electrochemical nanosensor platform on the GCE surface was designed by combining the conventional chemical approaches with the nanostructure-driven fabrication techniques and multiwalled carbon nanotubes (MWCNTs)/iron(III) oxide nanoparticles (Fe_2_O_3_ NPs)-chitosan nanocomposite (NC)/GCE sensor has been proposed for AXI determination. It has been well-accepted that MWCNTs are among the most preferred carbon-based nanomaterials for electrode modification due to their stability, excellent mechanical and conductive properties, low cost, and high surface area.^19,21−23^ In addition, Fe_2_O_3_ NPs can improve electron transfer kinetics, high surface area, biocompatibility, and nontoxic profile, and they are used to improve the catalytic performance of electrochemical systems.^23,27^ In addition, chitosan, as a natural polymer with high stability, biocompatibility, affordability, and ability to construct film matrixes, is widely preferred to be employed in combination with various nanomaterials.^27,28^ Taken together, due to the unique electrical and optical properties of MWCNTs, Fe_2_O_3_ NPs, and chitosan, a new electrochemical nanosensor platform (MWCNT/Fe_2_O_3_@chitosan NC) was fabricated based on the synergistic effect of MWCNTs, and Fe_2_O_3_ NPs dispersed in chitosan NC. The developed MWCNTs/Fe_2_O_3_@chitosan NC/GCE sensor was successfully applied to determine AXI in tablet dosage form and human serum samples with good accuracy. The results showed that MWCNTs/Fe_2_O_3_@chitosan NC/GCE sensor is an advantageous option for determining AXI with a low LOD value, high precision, and repeatability. Consequently, a nanoarchitectonics-based MWCNTs/Fe_2_O_3_@chitosan NC structure has been obtained with promising features for AXI sensing.

## Experimental Section

### Reagents and Chemicals

AXI and its tablet dosage form Inlyta were provided AXI and its tablet dosage form Inlyta was provided by DEVA Holding A.S. (Istanbul, Turkey). Acetic acid (≥99%), boric acid (≥99.5%), methanol (99.8%), phosphoric acid (>85%), sodium acetate trihydrate (>99%), sodium dihydrogen phosphate dihydrate (>99%), sodium hydroxide (>97%), sodium phosphate (96%), sodium phosphate monobasic (≥99%), sulfuric acid (95–97%), and drug-free human serum were obtained from Sigma-Aldrich. MWCNTs (>90% carbon basis, *D* × *L* 110–170 nm × 5–9 μm), Fe_2_O_3_ NPs (nanopowder, <50 nm particle size), and chitosan were also supplied from Sigma-Aldrich.

A 1 × 10^–3^ M AXI standard stock solution was prepared in methanol and kept in a refrigerator at 4 °C. Working solutions containing 20% methanol were prepared by dilution from the AXI stock solution with the supporting electrolyte solution. Buffer solutions of sulfuric acid solutions (pH 0.3–1.0), phosphate buffer solutions (pH 1.5–8.0), Britton–Robinson (BR) buffer solutions (pH 2.0–8.0), and acetate buffer solutions (pH 3.7–5.7) were prepared in double-distilled water and kept in a refrigerator at 4 °C.

### Equipment

All electrochemical measurements were performed using an AUTOLAB potentiostat/galvanostat (Nova 2.1.5 software, Netherlands). The three-electrode electrochemical cell system was constructed with a working electrode (GCE, 3 mm diameter), a reference electrode (Ag/AgCl electrode), and a counter electrode (Pt wire electrode). A pH meter from Mettler-Toledo (pH/ion S220, Switzerland) was utilized for pH measurement and adjustments. An electronic precision balance from Ohaus Instruments (Shanghai, China) was used to weigh the required chemicals.

### Preparation of MWCNTs/Fe_2_O_3_@chitosan NC/GCE

Before electrode modification, the GCE was ultrasonicated in a methanol/distilled water (1:1) mixture for 15 min. After that, the electrode surface was cleaned with alumina slurry on a polishing pad and washed with distilled water. The MWCNT dispersion (1:1) was prepared in *N*,*N*-dimethyl formamide. Chitosan was dispersed in 1% (v/v) acetic acid solution, and 2 mg of Fe_2_O_3_ NPs were dispersed in 2 mL of prepared chitosan mixture (0.1%, w/v). Five microliters of the MWCNT dispersion was dropped onto the GCE surface and dried in the vacuum oven for 15 min. This process was repeated two more times. After that, 0.5 μL of the Fe_2_O_3_ NPs-chitosan mixture was dropped onto the GCE surface three times with drying time intervals of 15 min in a vacuum oven. Before the electrochemical measurements, the prepared MWCNTs/Fe_2_O_3_@chitosan NC/GCE was activated electrochemically by 15 cycles of cyclic voltammetry (CV) between −0.2 and 1.6 V in a 0.1 M H_2_SO_4_ solution.

### Optimization of the Analytical Procedures

Differential pulse voltammetric (DPV) and adsorptive stripping DPV (AdSDPV) measurements were performed under the following optimum parameters: modulation amplitude of 0.05 V, modulation time of 0.05 s, step potential of 0.008 V, scan rate of 0.0159 V s^–1^, and equilibrium time of 5 s. For AdSDPV measurements, optimum stripping conditions were found as 0 V of accumulation potential and 60 s of accumulation time. CV measurements were performed under the following optimum parameters: scan rate of 0.5 V s^–1^, step potential of 0.00244 V, and interval time of 0.0244 s.

For applications in tablet dosage form and human serum samples, five tablets of Inlyta containing 5 mg of AXI were weighed. Then, the tablets were pulverized in a mortar until a homogeneous powder was obtained. A 1 × 10^–3^ M stock tablet solution was prepared in methanol using the required amount of tablet powder. Working solutions containing 20% methanol were prepared by diluting the stock tablet solution with a 0.1 M H_2_SO_4_ solution.

To prepare a 1 × 10^–4^ M M stock serum solution, 5.4 mL of acetonitrile, 3.6 mL of serum, and 1 mL of AXI stock solution were mixed in a centrifuge tube. The mixture was centrifuged for 20 min at 5000 rpm. After that, the supernatant part was separated and used to prepare working solutions containing 20% methanol with required dilutions using a 0.1 M H_2_SO_4_ solution.

### Quantum Mechanical Calculations

One of the possible interactions of analyte solution containing AXI with the modified surface is with chitosan, which is a polycationic polymer in the acidic medium at pH < p*K*_a_ = 6.5. It bears two hydroxyl groups and one amine group per repeating unit. These functional groups make chitosan very reactive against chemical modifications in solid state and also in solution.^29^ The chitosan as a part of NC forms strong electrostatic interactions with the MWCNT through the functional groups. It may serve as a supporting matrix for the Fe_2_O_3_ nanoparticles and binds noncovalently to AXI through H-bond interactions. It improves electron transfer between the electrode surface and the solution where the AXI is oxidized and detected electrochemically.

The mechanism of physical adsorption of AXI onto the electrode surface was studied by the density functional theory (DFT) method. The calculations at B3LYP/6-311+G(d,p) level^30^ were carried out for geometry optimizations and frequency calculations of AXI, glucosamine dimer (GD), as a unit representing chitosan, and their H-bonded complex. Some of the geometry optimizations were carried out in methanol solvent using a polarizable continuum model (PCM).^31^ The nonbonded interactions between the molecules in complexes were studied at the M062x/6-31g/(d,p) level. To gain more insight into the physisorption phenomenon of the drug, the electronic and optical properties of AXI before and after being subjected to modified GCE in solution were studied. Highest occupied molecular orbital (HOMO), lowest unoccupied molecular orbital (LUMO) energies, HOMO–LUMO band gap (Δ*E*), UV absorption spectra, and electrostatic potential energy (ESP) surfaces were obtained. The GCE has a microstructure similar to fullerene, and it easily accommodates MWCNT particles probably via π–π stacking type electrostatic interactions. The nanocomposite material is modeled as follows: The MWCNT was represented by a coronene surface. Fe_2_O_3_ nanoparticles were modeled according to their crystal structures in which each iron atom was hexagonally coordinated to oxygen atoms and shared three oxygens. The interaction of Fe_2_O_3_ with chitosan occurred via H-bonding through −NH_2_ (chitosan) and O (Fe_2_O_3_). To simply model the nanocomposite system on the GCE, the molecule with the [glucosamine dimer-Fe_2_O_3_-glucosamine dimer] sequence was physically adsorbed onto the coronene surface. The electron transfer rate and charge mobility^32^ were calculated for the coronene–coronene nonbonded interaction and compared with coronene–Fe_2_O_3_@chitosan nonbonded interaction.

## Results and Discussion

### Evaluation of Electrochemical Behavior by Electrochemical Impedance Spectroscopy (EIS)

**Figure 1:**
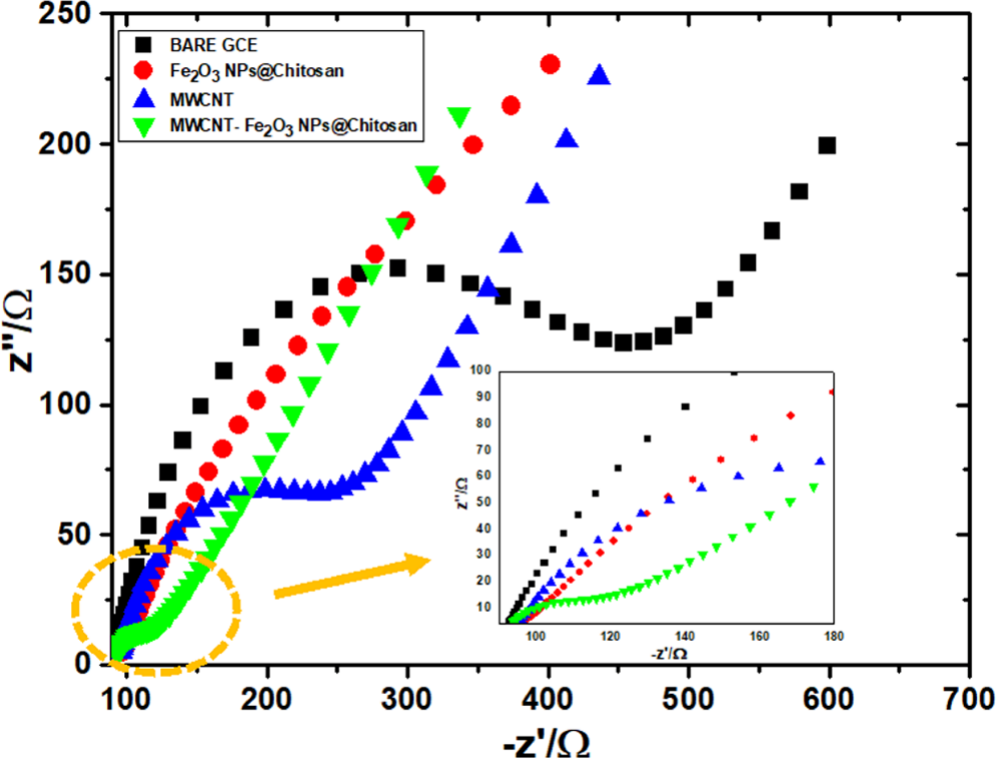
Nyquist plots: bare GCE (black), Fe_2_O_3_@chitosan NC (red), MWCNTs (blue), and MWCNTs/Fe_2_O_3_@chitosan NC (green) modified GCE in 5 mM [Fe(CN)_6_]^3–/4–^ solution. Inset: Randle equivalent electrical circuit.

The electrochemical impedance spectroscopy (EIS) technique was used to study the electrochemical behavior of bare and modified electrode surfaces. In EIS measurements, the open-circuit potential was 0.207 V, the frequency applied was between 0.1 and 1 × 10^5^ Hz, a 5 mM [Fe(CN)_6_]^3–/4–^ solution was used as a redox probe, and the measurement results were demonstrated as Nyquist plots. In a Nyquist plot, the semicircle region was related to charge-transfer resistance (*R*_ct_), and the *R*_ct_ value was calculated using this region’s diameter. The *R*_ct_ value was obtained by fitting the data using the equivalent circuit (Figure 1, inset). Higher *R*_ct_ values indicate that the electron transfer rate on the GCE surface is lower and slower. The enhancement of the electron transfer and the catalytic effects of the used nanomaterials were evaluated using this technique. Figure 1 shows that the highest *R*_ct_ value was obtained for the bare GCE (435.62 Ω). In addition, the *R*_ct_ values of Fe_2_O_3_@chitosan NC/GCE (312.56 Ω) and MWCNTs/GCE (221.60 Ω) were lower than those of the bare GCE, confirming the electroconductive and catalytic effect of nanomaterials on electron transfer. The lowest *R*_ct_ value was obtained for MWCNTs/Fe_2_O_3_@chitosan NC/GCE (35.57 Ω), confirming the synergistic effect of the NC and an enhanced and easier electron transfer on the surface.

### Surface Characterization of MWCNTs/Fe_2_O_3_@chitosan NC/GCE

**Figure 2:**
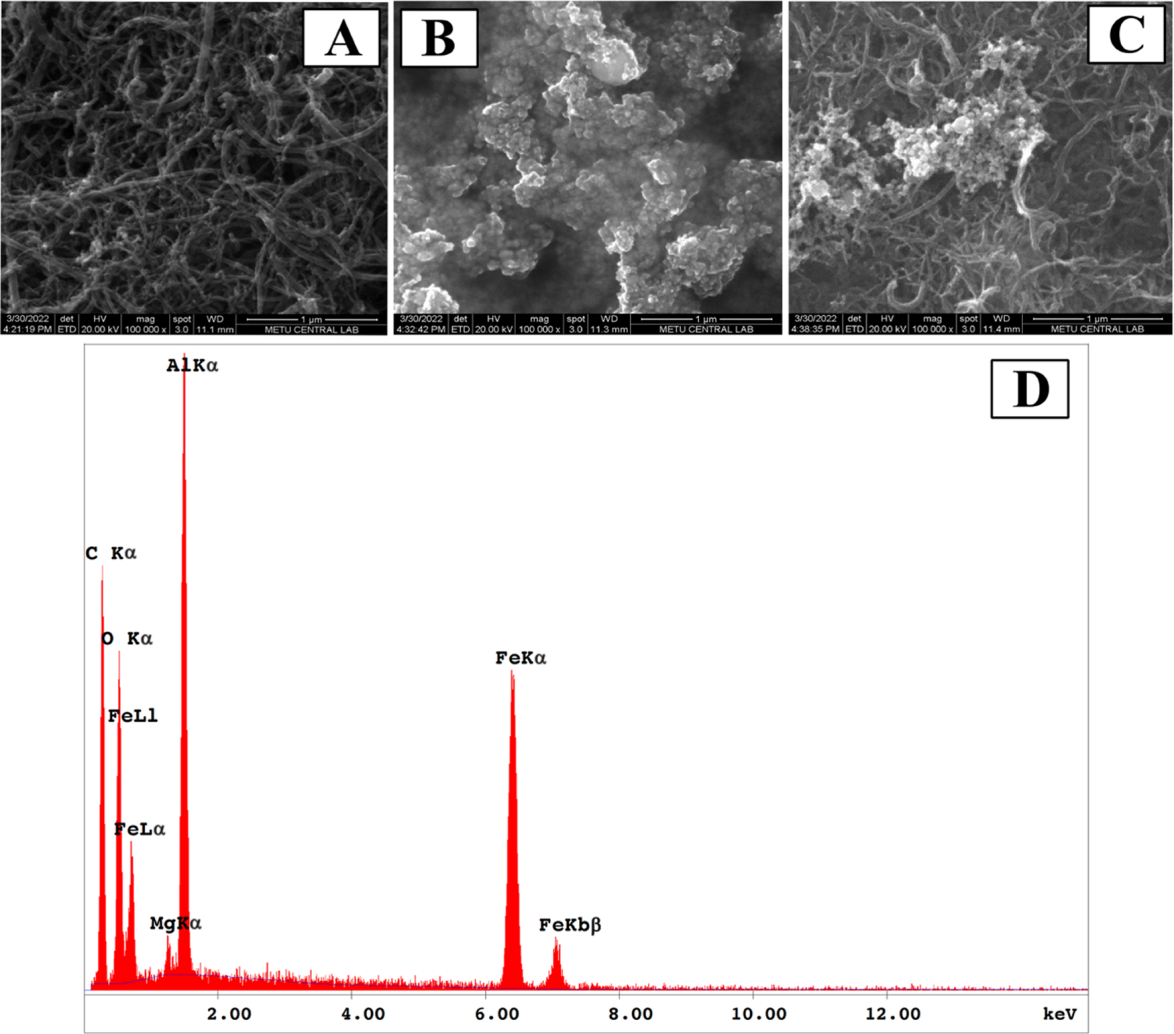
SEM images of (A) MWCNTs, (B) Fe_2_O_3_/chitosan NCs, and (C) MWCNTs/Fe_2_O_3_@chitosan NCs. (D) EDX spectra of MWCNTs/Fe_2_O_3_@chitosan NCs.

Surface morphological characteristics of MWCNTs/Fe_2_O_3_@chitosan NC/GCE were evaluated using scanning electron microscopy (SEM) and SEM energy-dispersive spectrometry (SEM-EDX). Figure 2 shows the surface characteristics of MWCNTs, Fe_2_O_3_@chitosan NC, and MWCNTs/Fe_2_O_3_@chitosan NC obtained with SEM and EDX spectra of MWCNTs/Fe_2_O_3_@chitosan NC. In Figure 2A, it can be seen that MWCNTs formed a three-dimensional homogeneous structure on the GCE surface that enables the active area on the surface. Figure 2B shows the obtained aggregated surface image of Fe_2_O_3_@chitosan NC due to the presence of chitosan. Using a mixture of MWCNTs/Fe_2_O_3_@chitosan NC (Figure 2C) enhanced the electroconductivity, active surface area, and electron transfer on the surface, resulting in a higher signal response of AXI. EDX spectra (Figure 2D) confirmed the presence of MWCNTs and Fe_2_O_3_@chitosan NC on the GCE surface.

### Effect of pH on the Electrochemical Behavior of AXI

**Figure 3:**
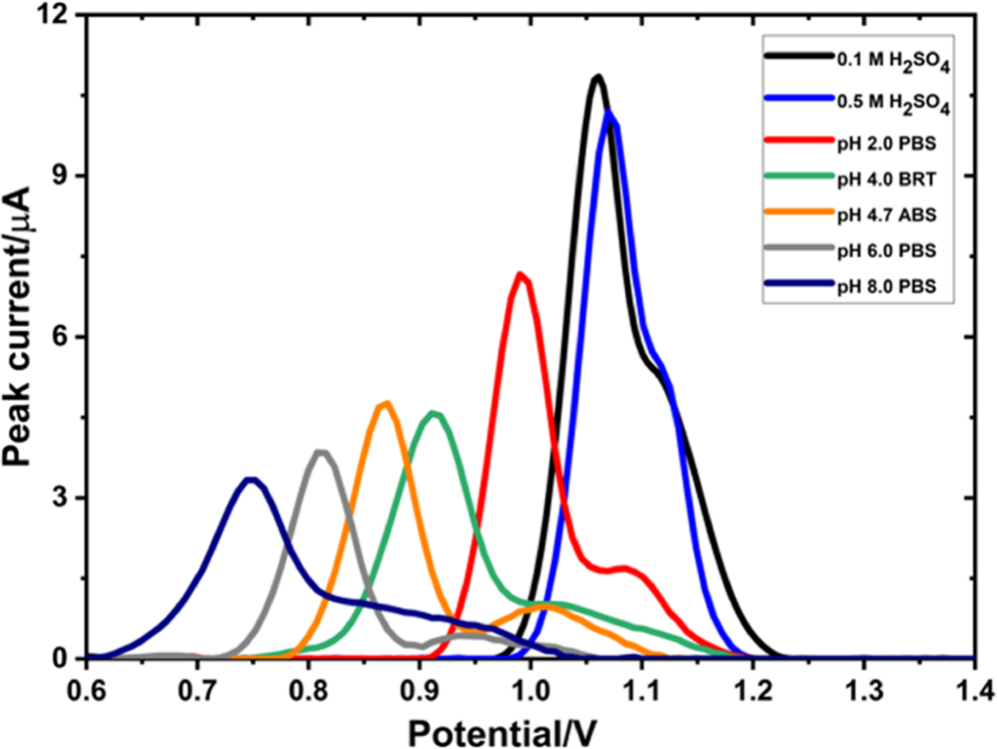
DPVs of AXI (40 μM) recorded at different pH values as 0.1 M H_2_SO_4_ (black), 0.5 M H_2_SO_4_ (blue), pH 2.0 phosphate buffer (red), pH 4.0 BR buffer (green), pH 4.55 acetate buffer (orange), pH 6.0 phosphate buffer (gray), and pH 8.0 phosphate buffer (navy blue).

The effect of pH on the electrochemical behavior of AXI was examined on MWCNTs/Fe_2_O_3_@chitosan NC/GCE. Different buffer solutions of acetate, phosphate, Britton–Robinson, and H_2_SO_4_ were used in the pH range between 0.3 and 8. When the obtained DPV voltammograms (Figure 3) are examined, it can be seen that the highest peak current values are obtained at pH 0.3 and 1. On the contrary, it can be observed that the peak current values decrease considerably toward the basic pH values.

The relationship between peak potential (*E*_p_) and pH was also examined. In the electrooxidation reaction, slope values imply that the number of protons is equal to the number of electrons.^33^
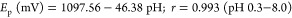
1

Consequently, a 0.1 M H_2_SO_4_ solution with the highest peak current value was chosen as the optimum pH value for further experiments.

### Effect of Scan Rate on the Electrochemical Behavior of AXI

The effect of scan rate was investigated to obtain information related to electrochemical processes and oxidation mechanisms. The electrochemical behavior of AXI on MWCNTs/Fe_2_O_3_@chitosan NC/GCE was evaluated in the range between 5 and 500 mV s^–1^ (Figure S1). It can be seen that the peak current (*I*_p_) values increased with the increasing scan rate (*v*) values. Additionally, the effect of *v* on the ip1 values of 2 × 10^–4^ M AXI in a 0.1 M H_2_SO_4_ solution has been examined (Figure S2). As seen in the equations below, ip1 was found to show a linear relationship with *v*.
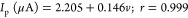
2
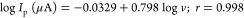
3

The square root of the scan rate (*ν*^1/2^) versus *I*_p_ graph showed a linear response, indicating the adsorption-controlled oxidation of AXI on MWCNTs/Fe_2_O_3_@chitosan NC/GCE. In addition, the slope value of the linear relationship of log *ν* versus log *I*_p_ was obtained as 0.79, confirming the adsorption-controlled oxidation mechanism.

### Effect of Experimental Conditions on the Preparation of MWCNTs/Fe_2_O_3_@chitosan NC/GCE

#### Amount of MWCNTs/Fe_2_O_3_@chitosan NC

**Figure 4:**
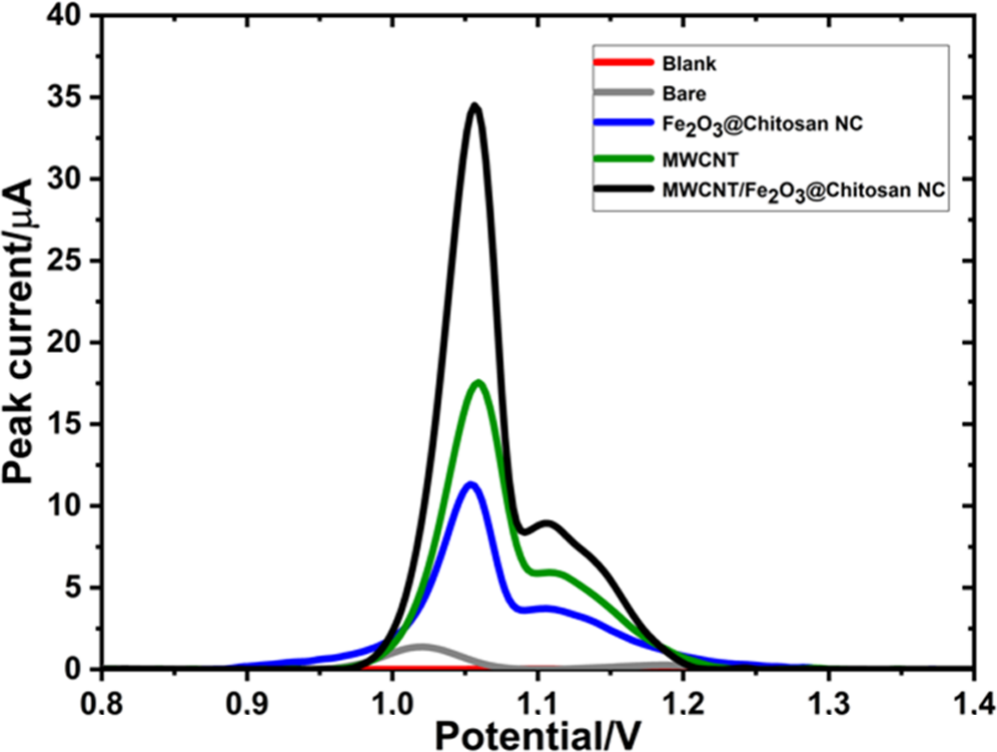
Influence of the amount of modifier on 40 μM solutions of AXI. (A) DPVs and (B) CVs were obtained for bare, MWCNT, Fe_2_O_3_@chitosan NC, and MWCNT/Fe_2_O_3_@chitosan NC modified GCE in 0.1 M H_2_SO_4_.

To determine the effect of the used nanocomposite amount on the electrochemical behavior of AXI, the peak currents of 4 × 10^–5^ M AXI obtained with DPV and AdSDPV were evaluated. The results obtained with both techniques corresponded to each other, and since the peak currents were higher, they were evaluated over AdSDPV. An approximately 12-fold increase in the peak current of AXI was observed with the GCE modified with MWCNTs, which was prepared by dropping 5 μL of nanomaterial three times by a sandwich method. When the Fe_2_O_3_@chitosan NC modified GCE was prepared in the same way, a sevenfold increase was obtained. In addition to that, to observe the synergistic effect of these two different nanomaterials, 0.5, 1, 1.5, 2, and 3 μL of Fe_2_O_3_@chitosan NC were dropped onto the MWCNTs/GCE surface, and the peak currents were evaluated. As a result, the highest increase in the peak current (approximately 23-fold) was obtained with 1.5 μL of nanocomposite and used as the optimum procedure for sensor preparation (Figure S3). However, the results showed that the maximum current increase was obtained due to the synergistic effect of MWCNTs/Fe_2_O_3_@chitosan nanocomposite modified electrode components (Figure 4). The modified sensor captured AXI within its porous structures and provided more attachment sites. Thus, it significantly contributed to more AXI deposition on the modified GCE surface. Additionally, the prepared nanocomposite provides a catalytic effect to increase the electron transfer rate on the surface. This results in an enhanced electrooxidation performance of the sensor and increased peak current values.

#### Accumulation Potential and Time

After confirming the adsorption-controlled oxidation mechanism of AXI on MWCNTs/Fe_2_O_3_@chitosan NC/GCE, accumulation potential (*E*_acc_) and accumulation time (*t*_acc_) parameters of AdSDPV were optimized. First, the peak current responses of 5 × 10^–4^ M AXI were evaluated by AdSDPV in the range of 0–1.2 V (*t*_acc_ = 60 s) to observe the effect of *E*_acc_. Figure S4A shows that after the highest *I*_p_ value was obtained at 0 V, a constant decrease in *I*_p_ was observed. Therefore, time optimization was made at 0 V with the same AXI concentration. When the different times in the range of 0–240 s were evaluated, it was determined that the highest *I*_p_ value was acquired at 60 s and was used as the optimum value in the following experiments (Figure S4B).

### Evaluation of the Analytical Performance

**Figure 5:**
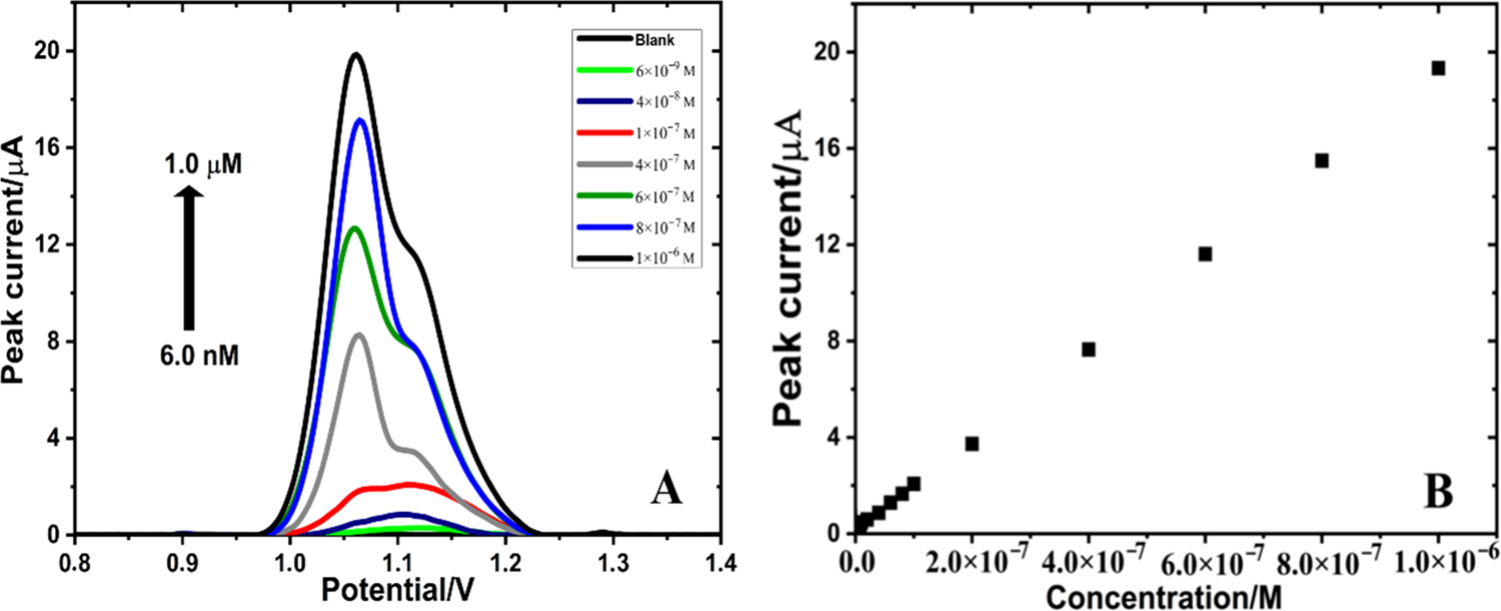
(A) AdSDPV obtained at MWCNTs/Fe_2_O_3_@chitosan NC/GCE for AXI (in 0.1 M H_2_SO_4_ solution) at different concentrations. Inset (B) is the calibration plot for AXI.

The analytical performance of the MWCNTs/Fe_2_O_3_@chitosan NC/GCE sensor was evaluated under the optimum experimental conditions. AdSDPV measurements were performed to determine AXI in the linear concentration range between 6 × 10^–9^ and 1 × 10^–6^ M (Figure 5). The regression equation corresponding to the calibration curve was found to be *I*_p_ (μA) = 1.92 × 10^6^*C* (M) + 0.147783 (*R*^2^ = 0.999). Based on the standard deviation of the response and the slope,^34^ the limit of detection (LOD, LOD = 3*s*/*m*) and the limit of quantification (LOQ, LOQ = 10*s*/*m*) values were calculated to be 9.04 × 10^–11^ and 3.01 × 10^–10^ M, respectively. The obtained very low LOD and LOQ values, wide linear concentration range, and the high sensitivity of the MWCNTs/Fe_2_O_3_@chitosan NC/GCE sensor demonstrated the advantages of this method compared to other available studies for AXI determination. Table 1 summarizes the regression data of the calibration line for AXI and emphasizes the good repeatability and reproducibility results of the developed sensor.

**Table 1:** Regression Data of the Calibration Line for AXI on MWCNTs/Fe_2_O_3_@chitosan NC/GCE

	standard solution	human serum sample
linearity range (M)	(6 × 10^–9^)–(1 × 10^–6^)	(6 × 10^–9^)–(1 × 10^–6^)
slope (μA M^–1^)	1.912 × 10^7^	2.071 × 10^7^
SE of slope	8.790 × 10^4^	1.728 × 10^5^
intercept (μA)	0.13942	–0.0617
SE of intercept	0.03634	0.07147
correlation coefficient (*r*)	0.999	0.999
LOD (M)	9.04 × 10^–11^	1.44 × 10^–10^
LOQ (M)	3.01 × 10^–10^	4.83 × 10^–10^
repeatability of peak current (RSD%)^a^	0.269	0.754
reproducibility of peak current (RSD%)^a^	1.713	1.961

a Each value is the mean of three experiments.

There are only two other electrochemistry-based studies on the literature for the determination of AXI (Table 2). Cetinkaya et al.^2^ evaluated the electrochemical behavior of AXI on GCE and boron-doped diamond electrode (BDDE). The other study is based on a molecularly imprinted polymer (MIP) sensor.^35^ The MWCNTs/Fe_2_O_3_@chitosan NC/GCE sensor is more sensitive and has lower LOD values compared to the bare electrodes. Even though the MIP-based sensor has lower LOD values and good selectivity, this present work offers better stability and a better understanding of the electron transfer mechanism on the GCE surface with the density functional theory.

**Table 2:** Comparison of Other Electrochemistry-Based Studies on the Determination of AXI with the Present Work

sensor	linearity range (M)	LOD (M)	LOQ (M)	ref
GCE and BDDE	(8 × 10^–8^)–(2 × 10^–6^) (GCE)	1.11 × 10^–9^ (GCE)	4.09 × 10^–9^ (GCE)	(2)
	(6 × 10^–7^)–(8 × 10^–5^) (BDDE)	4.09 × 10^–8^ (BDDE)	1.36 × 10^–7^ (BDDE)	
MIP@*o*-PD/GCE	(1 × 10^–13^)–(1 × 10^–12^)	2.70 × 10^–14^	8.90 × 10^–14^	(35)
MWCNTs/Fe_2_O_3_@chitosan NC/GCE	(6 × 10^–9^)–(1 × 10^–6^)	9.04 × 10^–11^	3.01 × 10^–10^	this work

### Determination of AXI in Tablet Dosage Form and Human Serum Samples

**Figure 6:**
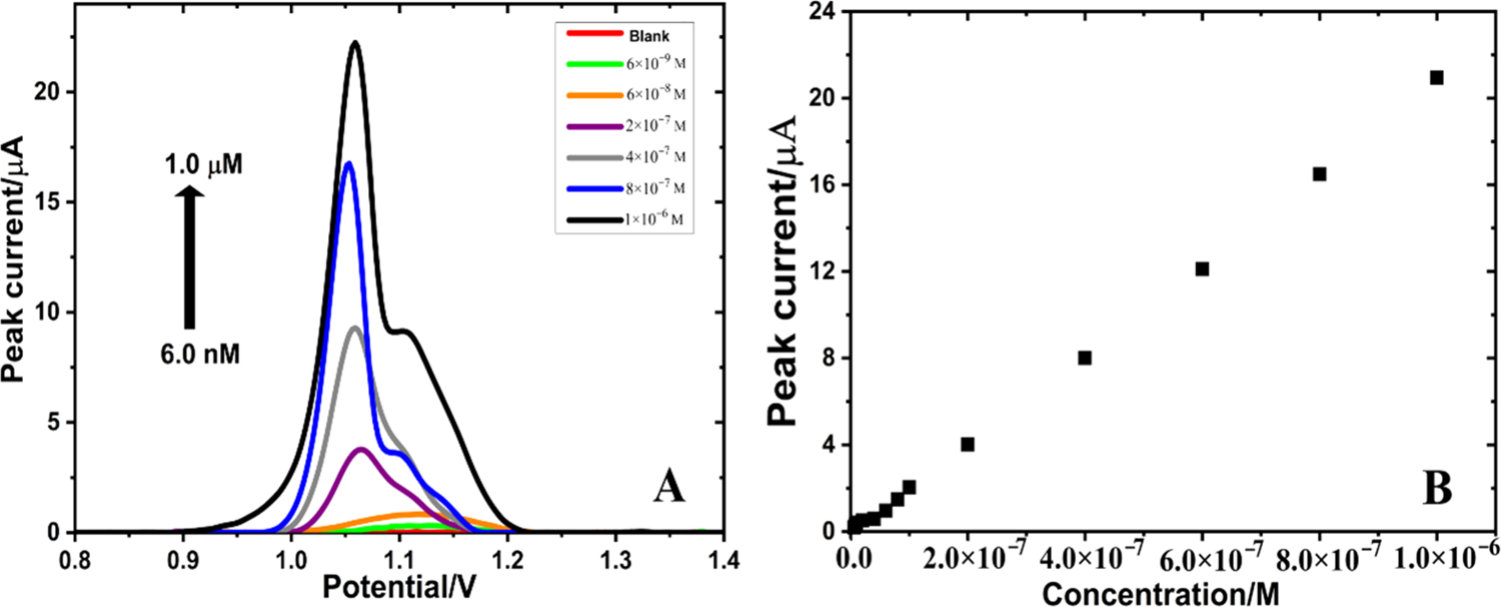
(A) AdSDPV obtained at MWCNTs/Fe_2_O_3_@chitosan NC/GCE for AXI (in 0.1 M H_2_SO_4_ solution) at different concentrations in the serum samples. Inset (B) is the calibration plot for AXI.

Accuracy and applicability of the MWCNTs/Fe_2_O_3_@chitosan NC/GCE sensor were evaluated on the tablet dosage form and human serum samples with recovery studies. The spiked serum samples were prepared as explained in Quantum Mechanical Calculations Section, and the calibration curve for AXI determination in serum samples gave a linear response with the regression equation of *I*_p_ (μA) = 1.92 × 10^6^*C* (M) + 0.147783 (*R*^2^ = 0.999) in Figure 6. The LOD and LOQ values and other analytical parameters are given in Table 3. Furthermore, recovery studies were performed for biological and pharmaceutical samples using the standard addition method. Excellent recovery results (between 99.23 and 101.84%) were obtained, proving that the MWCNTs/Fe_2_O_3_@chitosan NC/GCE sensor is an accurate, precise, and reliable option for AXI analysis.

**Table 3:** Results of the Tablet Dosage Form and Recovery Experiments

	tablet dosage form (inlyta)	serum sample
labeled claim (mg)	5.00	
amount found (mg)	4.98	
RSD%	1.39	
bias%	–0.6	
added (mg)	0.500	0.500
found (mg)	0.496	0.509
average recovery%	99.23	101.84
RSD% of recovery^a^	1.09	1.88
bias%	–0.77	1.84

a Each value is the mean of three experiments.

### Interference Study

**Figure 7:**
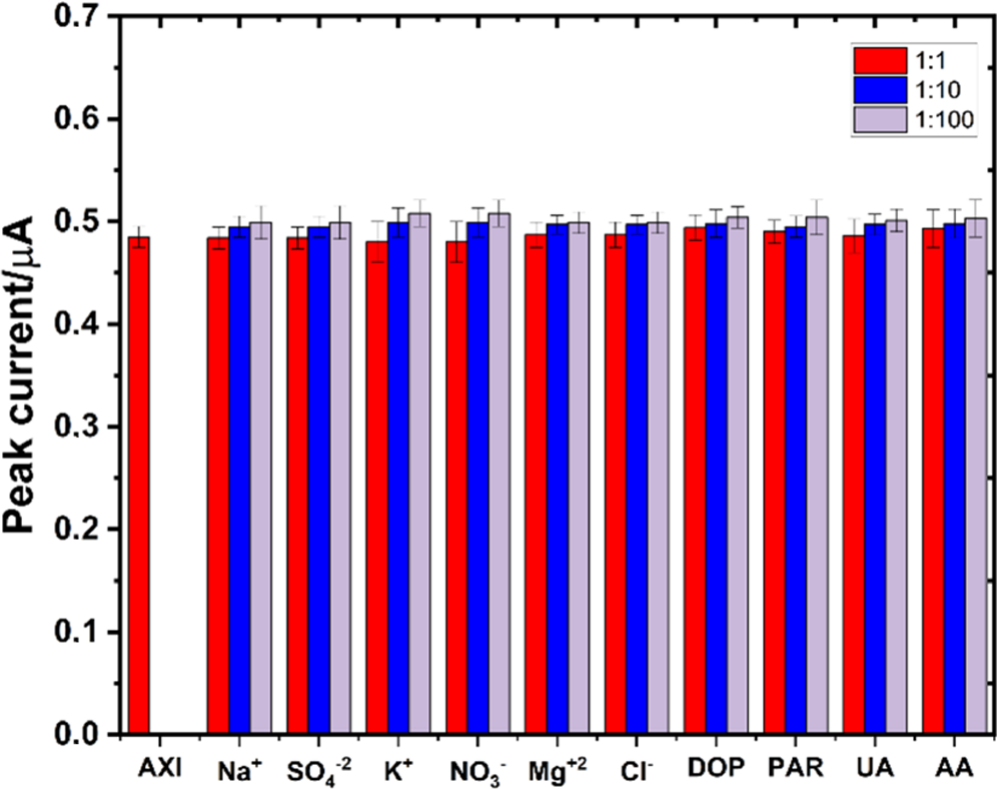
Bar graphs of peak currents of 20 nM AXI at 0.1 M H_2_SO_4_ with MWCNTs/Fe_2_O_3_@chitosan NC/GCE in the presence of interfering agents: Na^+^, SO_4_^2–^, K^+^, NO_3_^–^, Mg^2+^, Cl^–^, DOP, PAR, AA, and UA.

Interference studies were performed to show selectivity and the interference-free performance of the MWCNTs/Fe_2_O_3_@chitosan NC/GCE sensor. For this purpose, the most common interfering agents of Na^+^, SO_4_^2–^, K^+^, NO_3_^–^, Mg^2+^, Cl^–^, dopamine (DOP), paracetamol (PAR), ascorbic acid (AA), and uric acid (UA) were tested as mixtures with AXI at different molar ratios of 1:1, 1:10, and 1:100. As given in Figure 7, recovery experiments resulted in recovery% values ranging between 98.2 and 103.85, showing that the peak current response of AXI was not affected by the selected interfering agents on MWCNTs/Fe_2_O_3_@chitosan NC/GCE. Good relative standard deviation (RSD)% values ranging between 0.91 and 1.98% were obtained.

### DFT Calculations

**Figure 8:**
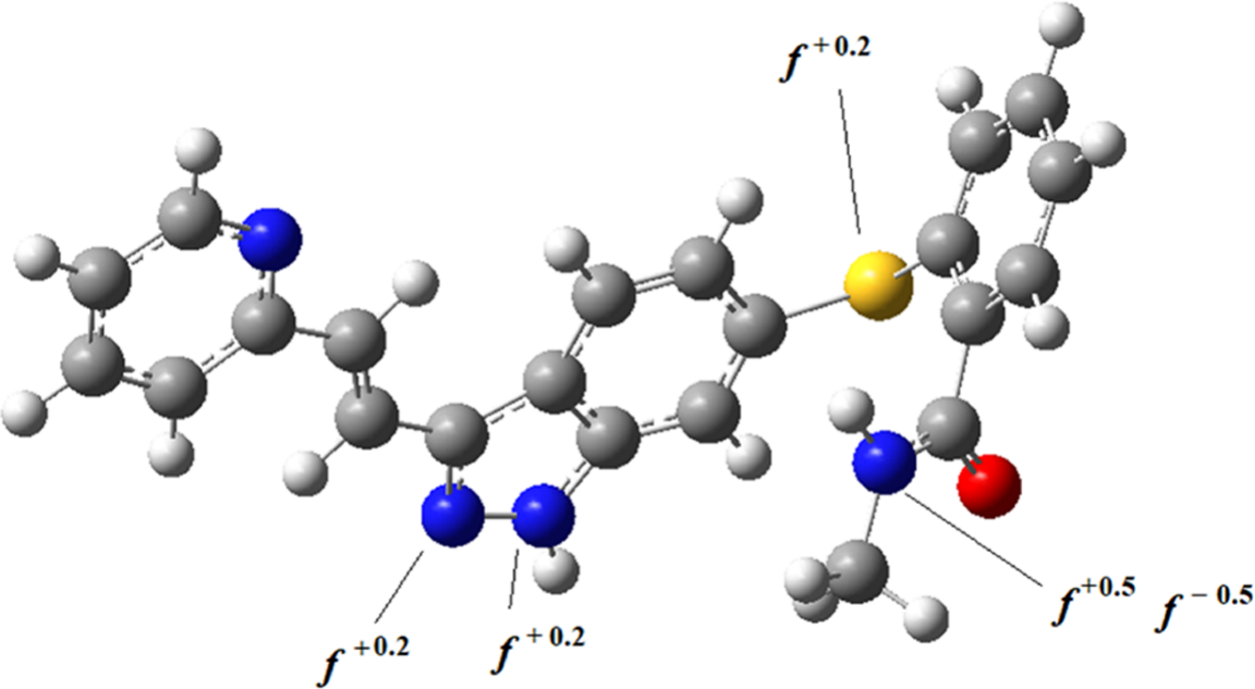
Optimized geometry of AXI in methanol. Fukui indices on the most reactive atoms are shown.

**Figure 9:**
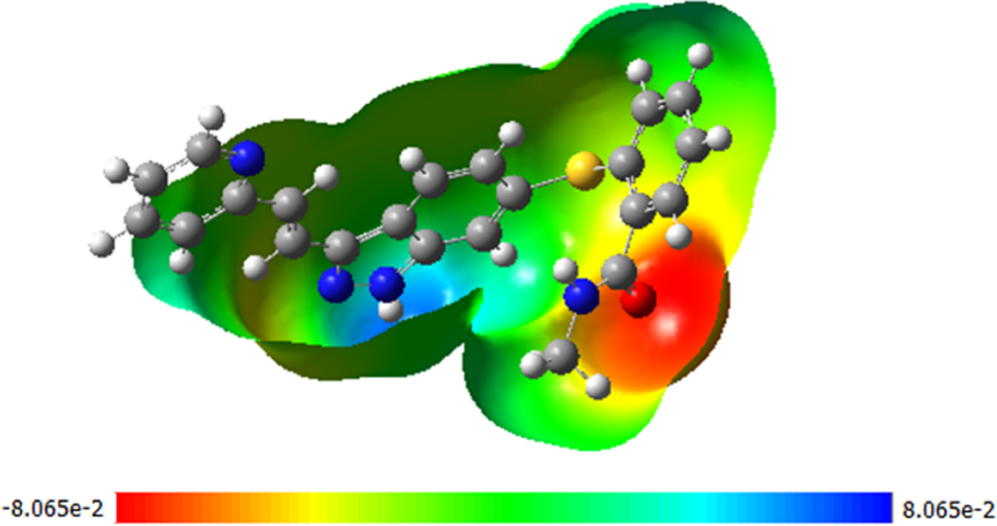
Electrostatic potential surface (ESP) (open top view) of the AXI molecule.

In Figure 8, the optimized geometry of neutral AXI molecule using Becke-3-parameter-Lee-Yang-Parr hybrid functional (B3LYP) and 6-311+G(d,p) basis sets in methanol solution is given. The calculated Fukui indices^36^ on the reactive atoms for nucleophilic (*f*^+^) and electrophilic (*f*^–^) interactions were shown. The nitrogen in the methyl amino group was found to be the most reactive atom in the molecule in methanol. The charge density is localized on the heteroatoms of the molecule, mostly on the oxygen and the nitrogen atoms as shown in the electrostatic potential energy (ESP) surface (Figure 9).

**Figure 10:**
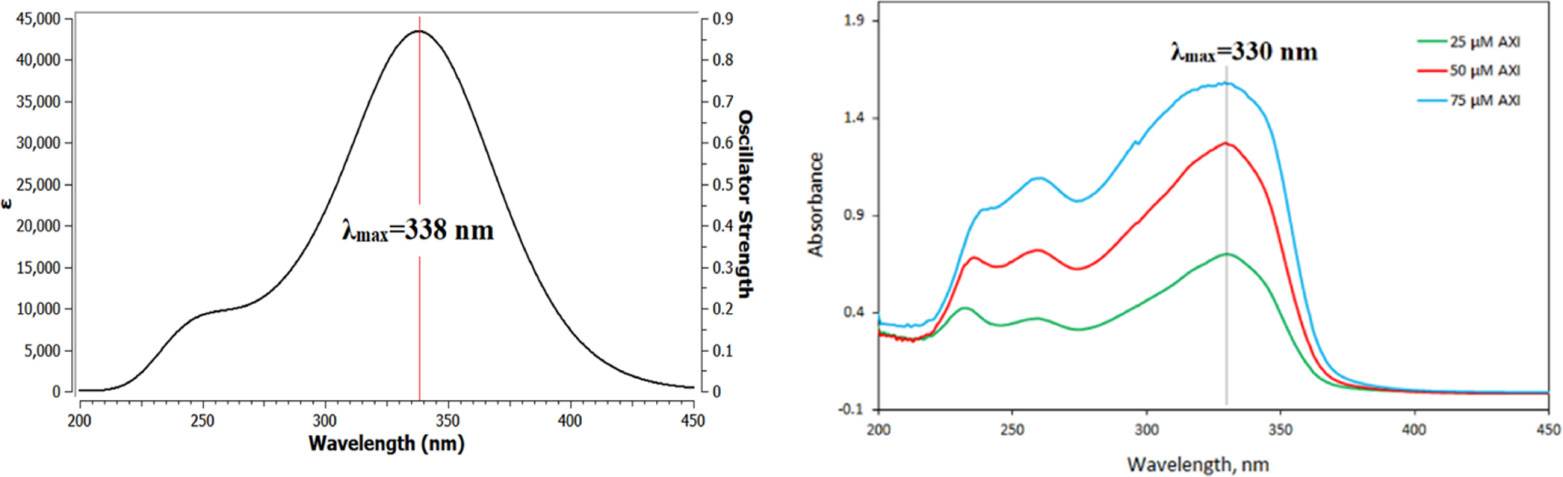
Theoretical (left) and experimental (right) UV–vis spectra of AXI in methanol.

The aromatic ring nitrogens and the sulfur atom of the phenyl sulfonyl moiety may have some potency as H-bond acceptors. After the drug AXI was dissolved in methanol solvent, its experimental UV–vis spectrum was obtained. For the validation of our DFT methodology, we compared theoretical and experimental spectra (Figure 10). The maximum absorption wavelength for π → π* (or HOMO → LUMO) transition was observed at 338 nm (theoretical) and 330 nm (experimental), indicating that the optimized geometries in solvent are reliable for further calculations.

**Figure 11:**
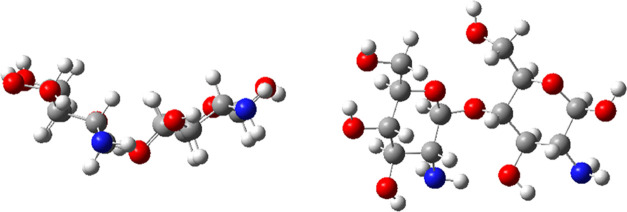
Optimized geometry of glucosamine dimer (GD) in methanol: side view (left) and top view (right).

Chitosan bears highly reactive functional groups, primary amino and hydroxyl groups, and secondary hydroxyl groups (Figure 11). Similarly, the AXI molecule also has reactive heteroatoms, pyridinic and pyrrolic nitrogens, carbonyl oxygen, and sulfur atom. According to the calculated Fukui indices for electrophilic and nucleophilic interactions, the heteroatoms are indeed the most active centers, especially the terminal −NH group in the amide moiety is found to be the most reactive center due to its H-bond acceptor and donor abilities.

**Figure 12:**
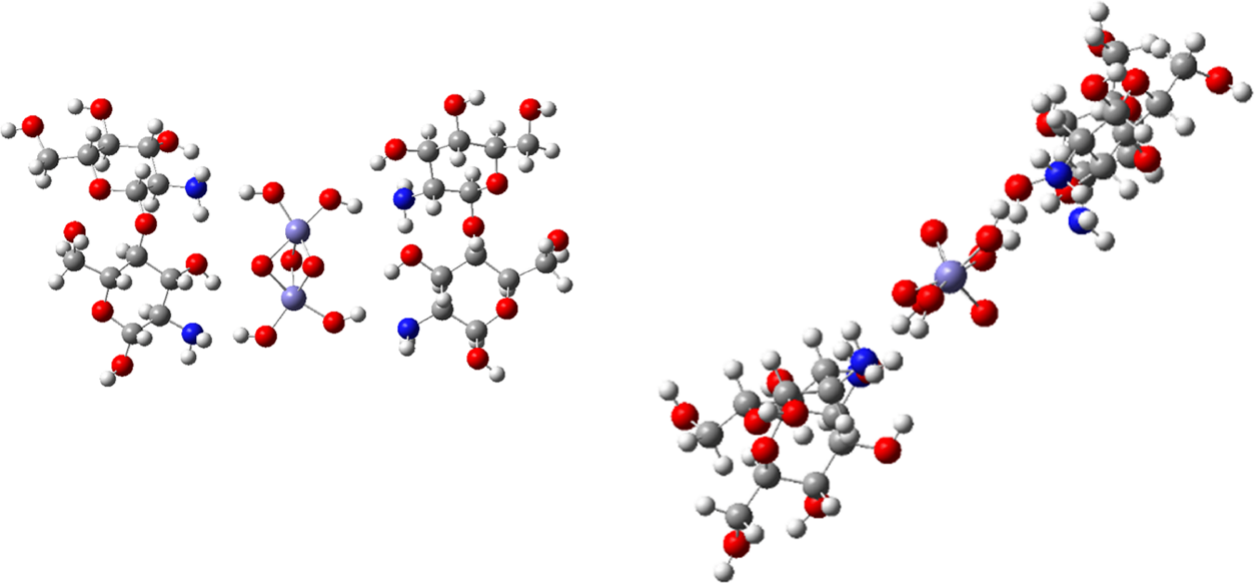
Optimized geometry of [glucosamine dimer–Fe_2_O_3_–(OH)_4_–glucosamine dimer] complex: top view (left) and side view (right).

When AXI is brought into contact with NC in solution, the expected and also dominating interaction between the AXI and chitosan molecules will be H-bond interactions. Chitosan is a highly basic molecule with pyrollic nitrogens whose p*K*_a_ is 6.5. At pH < p*K*_a_, amine groups readily abstract protons and become protonated, which makes the molecule cationic. For these reasons, the nonbonded interaction between AXI and chitosan is studied through a model system in which AXI establishes H-bond interactions with dicationic glucosamine dimer (Figure 12).

Because of the oxygen bridges connecting the repeating units, it is highly possible that the polymer chains become helical as the chain length increases and wrap the carbon nanotubes they interact with through strong electrostatic interactions.

**Figure 13:**
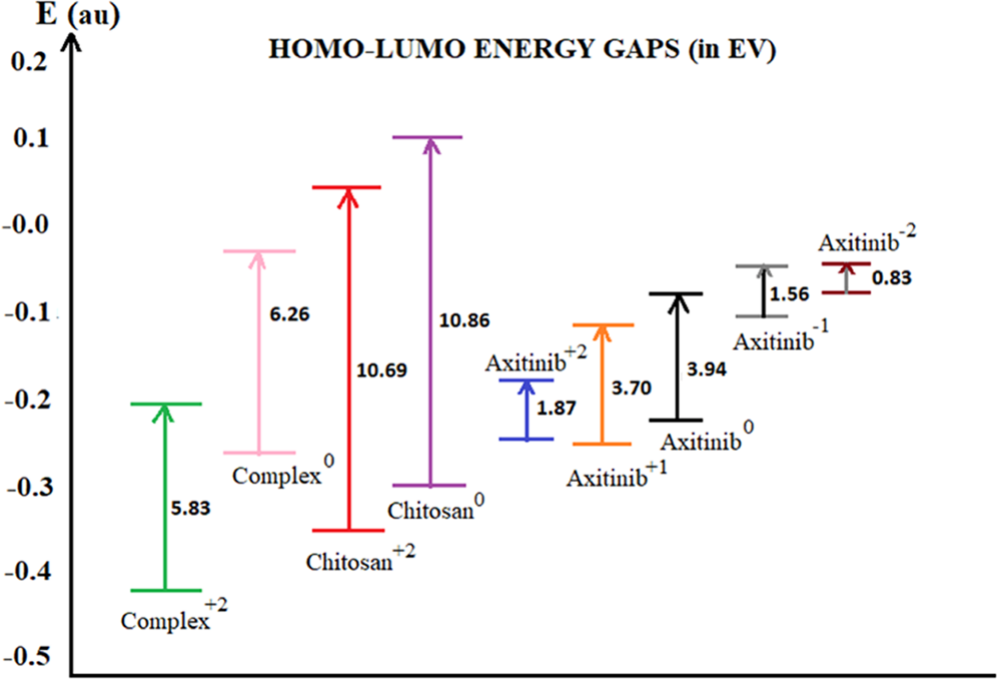
HOMO–LUMO energy gaps for AXI, chitosan, and their nonbonded complex at different charged states.

**Figure 14:**
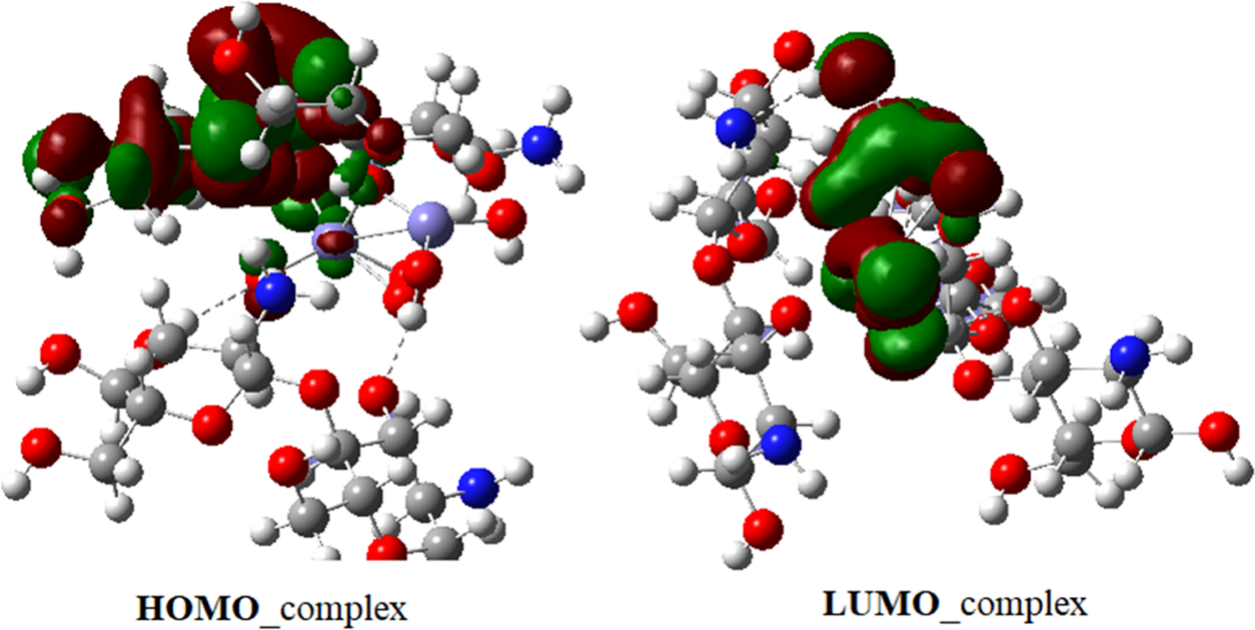
HOMO and LUMO of the [glucosamine dimer–Fe_2_O_7_–glucosamine dimer] system.

The changes in the HOMO–LUMO energy gap (Figure 13) and the localization of the frontier orbitals upon complexation (Figure 14) were examined.

While the HOMO is localized on the chitosan side, LUMO is localized on the iron oxide.

**Figure 15:**
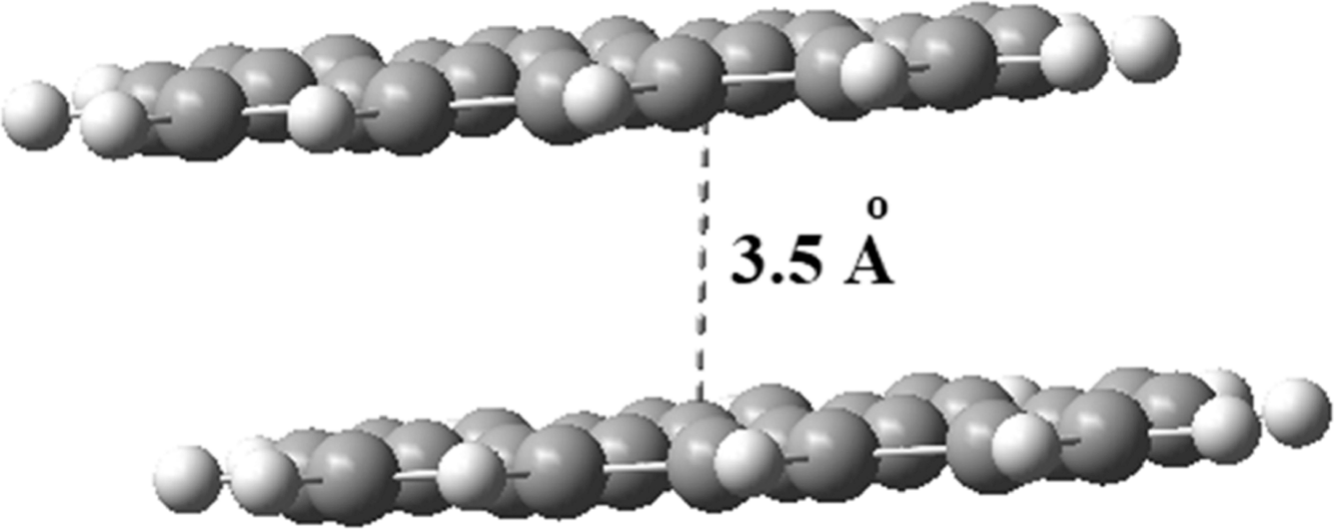
Side view of the coronene dimer.

The nonbonded interactions between two coronene sheets that are located on top of each other to allow π–π stacking interactions (Figure 15) were studied to compare the changes in the charge-transfer rate and the charge mobility when the upper surface was replaced by our iron oxide imprinted chitosan model system. The latter system suffers from scf convergence problems and needs further studies. However, the charge mobility (μ) and charge-transfer rate (*k*) for coronene dimer were calculated to be 0.26 cm^2^ V^–1^ s^–1^ and 1.08 × 10^13^ s^–1^, respectively, which were fairly good for such a small-size system. According to the experimental findings, it was expected that the Fe_2_O_3_@chitosan-enriched MWCNT surface has higher charge mobility and faster electron transfer kinetics when compared to an unmodified electrode.

**Figure 16:**
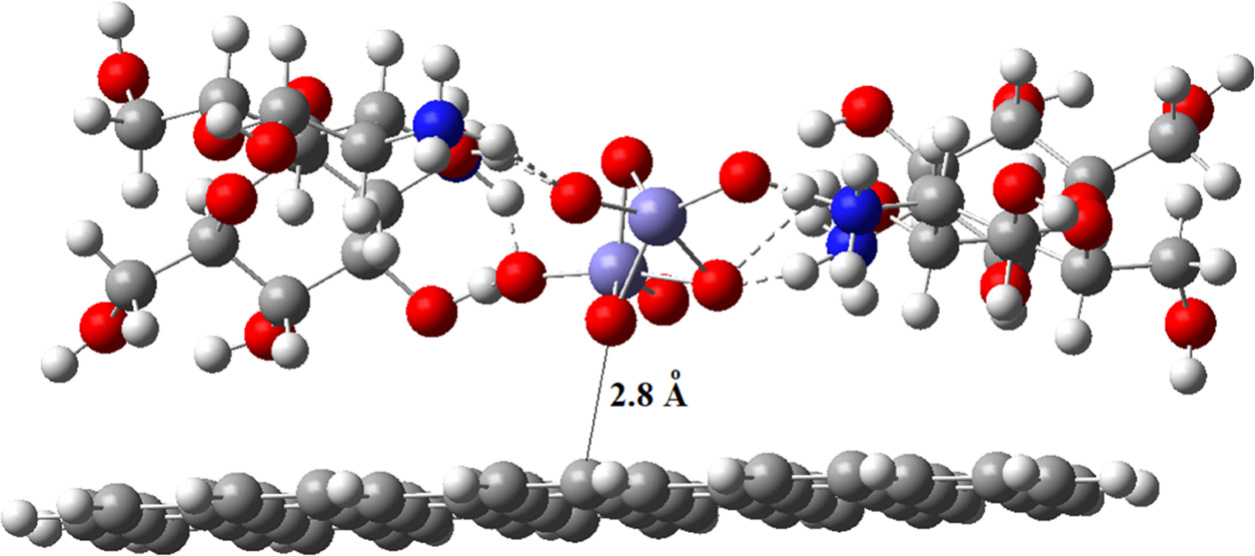
Model surface for Fe_2_O_3_@chitosan adsorbed on MWCNT. Dotted lines are the hydrogen bonds between NH_2_H···O and O–H–O (side view).

Our estimated interaction of the iron oxide nanoparticles stabilized by incorporation into the chitosan matrix will be with the surface of the MWCNTs, as shown in Figure 16. Although the distance of the closest atom from the surface was 2.8 Å, it changes from atom to atom and is energetically the most stable conformation of the Fe_2_O_3_@chitosan NPs on the surface in the solid state.

**Figure 17:**
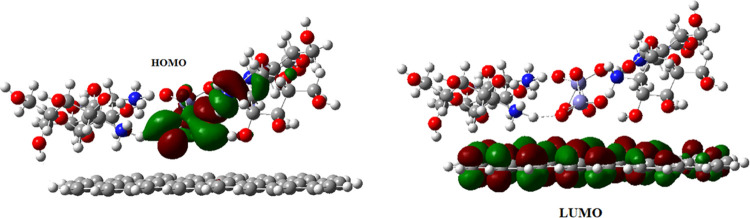
HOMO and LUMO of the Fe_2_O_3_@chitosan adsorbed on the MWCNT.

It was experimentally proved that the increased charge-transfer activity of the MWCNT-coated GCE surface stemmed from the presence of iron oxide nanoparticles since they channel charge from the solution to the electrode. During the oxidation process of AXI, the charge is transferred from the HOMO of the iron oxide moiety to the LUMO of the MWCNT (Figure 17). The negatively charged surface established strong electrostatic interactions including ionic bonds and H-bonding.

## Conclusions

This study explains the first nanosensor application to determine AXI in various samples. Modifying the GCE with MWCNTs and Fe_2_O_3_@chitosan NC provided a synergistic effect and enhanced the sensor performance by enabling the analysis of AXI at very low concentrations that cannot be obtained by bare GCE. Thus, very low LOD and LOQ values and accurate, precise, and reliable analyses were acquired with good repeatability and reproducibility compared to the bare electrode application. The developed sensor was also applied to the pharmaceutical tablet dosage form and human serum samples. The obtained wide linear concentration range of the MWCNTs/Fe_2_O_3_@chitosan NC/GCE sensor and excellent recovery results are advantageous for real-sample analysis. The MWCNTs/Fe_2_O_3_@chitosan NC/GCE sensor can be used with good stability for two days. Additionally, electrochemical characterization and surface characterization studies demonstrated the electrochemical behavior of the developed sensor surface. Interference studies confirmed the selectivity and interference-free performance of MWCNTs/Fe_2_O_3_@chitosan NC/GCE toward AXI.

Consequently, this study describes the first nanosensor application for the electrochemical assay of AXI in standard solution and biological and pharmaceutical samples in detail. In addition, the DFT calculations complemented excellent experimental results at the molecular level and shed light on the charge-transfer mechanism with the miniature models of the macroscopic system. We observed that the iron oxide nanoparticles form a bridge between the interacting species and play an important role in channeling the electron flow from the analyte solution to the electrode. The AXI detection mechanism depends on its efficient physisorption onto the modified electrode surface due to the noncovalent interactions between the AXI molecules and the NC in a solution.

## Supporting Information Available

Recycling experiment, details of powder X-ray diffraction (XRD) analysis, and copies of ^1^H and ^13^C NMR spectra of all products (PDF)
