# Supplementary material for: Detection of Axitinib Using Multiwalled Carbon Nanotube-Fe2O3/Chitosan Nanocomposite-Based Electrochemical Sensor and Modeling with Density Functional Theory
Source: ACS Omega. 2022 Sep 16;7(38):34495–505. doi: 10.1021/acsomega.2c04244 (PMC9520732; doi:10.1021/acsomega.2c04244)
Supplement: Supplementary file 1 — ao2c04244_si_001.pdf [file ao2c04244_si_001.pdf]

Supporting information

Detection of Axitinib Using Multiwalled Carbon Nanotube-Fe_2O_3/Chitosan Nanocomposite-Based Electrochemical Sensor and Modeling with Density Functional Theory

Ahmet Cetinkaya,[a] S. Irem Kaya,[a,b] Pelin Şenel,[c] Nejla Cini,[c] Esen B. Atici,[d] Sibel A. Ozkan,[a] Mine Yurtsever,[c,*] and Ayşegül Gölcü[c,*]

[a] Ankara University, Faculty of Pharmacy, Department of Analytical Chemistry, Ankara, Turkiye.

[b] University of Health Sciences, Gulhane Faculty of Pharmacy, Department of Analytical Chemistry, Ankara, Turkiye.

[c] Istanbul Technical University, Science and Letters Faculty, Chemistry Department, 34469, Maslak, Istanbul, Turkiye.

[d] DEVA Holding A.S., Research&Development Center, Tekirdağ, Turkiye.

*Corresponding Authors: aysgolcu@itu.edu.tr (Ayşegül Gölcü); mine@itu.edu.tr (Mine Yurtsever)

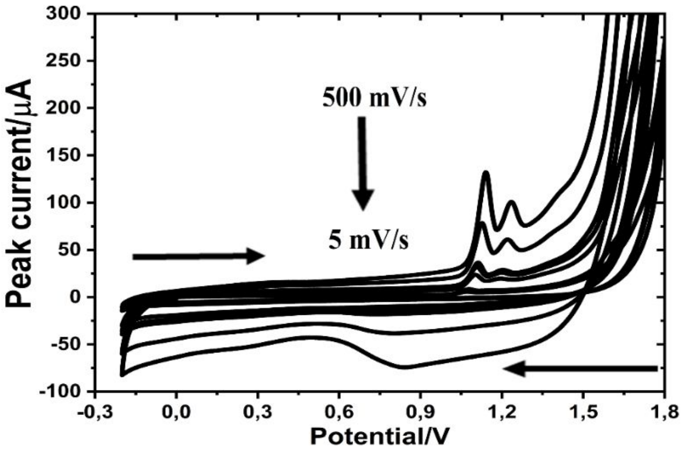

Figure S1. Cyclic voltammograms of 2×10^{-4} M AXI solutions in 0.1 M H_2SO_4 at various scan rates in the range between 5 and 500 mVs^{-1}.

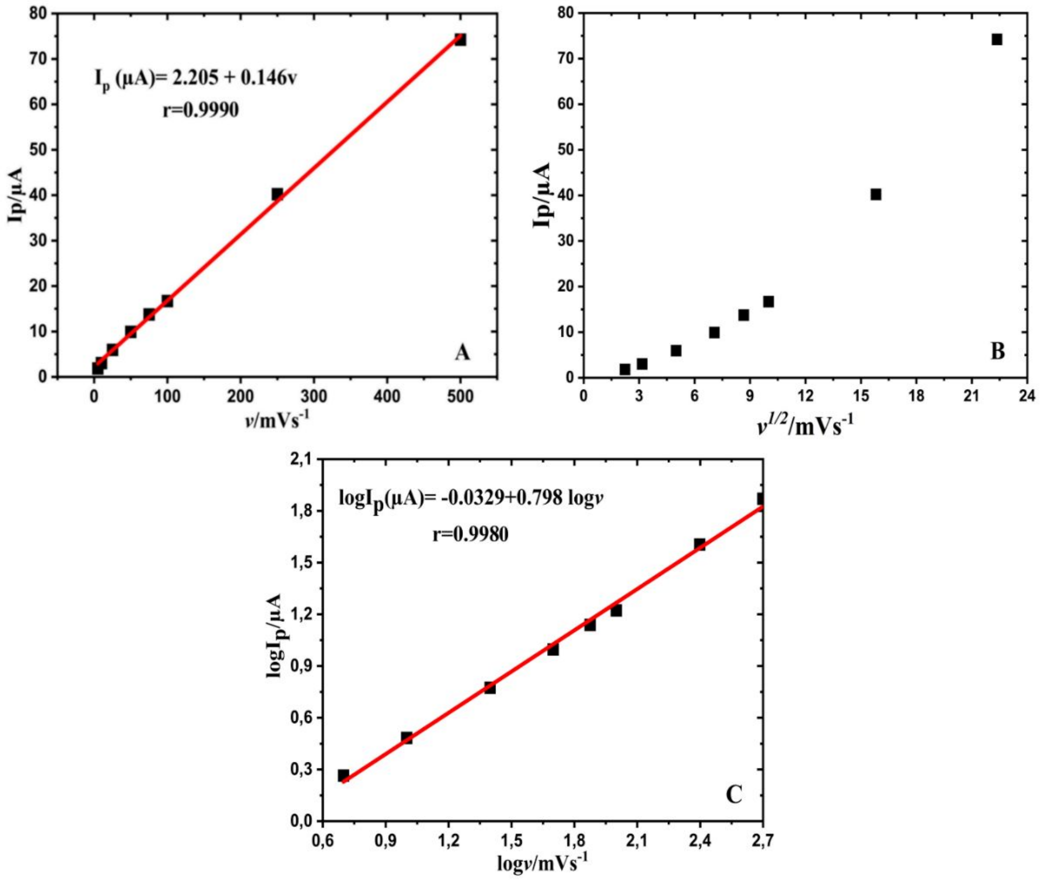

Figure S2. The plot of 2x10^{-4} M AXI: I_p versus v (A), I_p versus $v^{1/2}$ (B), and $\log I_p$ versus log$v$ (C).

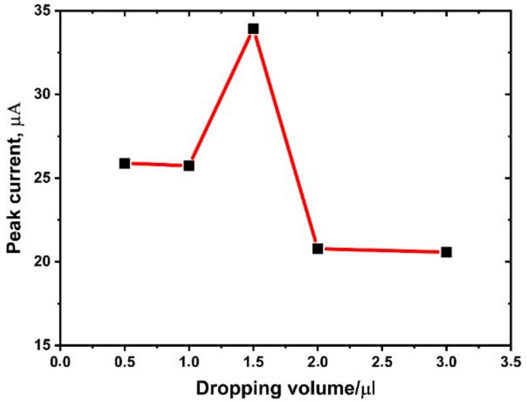

Figure S3. The plot of the DP voltammetric responses of 4×10^{-5} M DPG with different volume of modifier (MWCNTs/Fe_2O_3@Chitosan NC) suspension; 0.5 μL, 1 μL, 1.5 μL, 2 μL, and 3 μL.

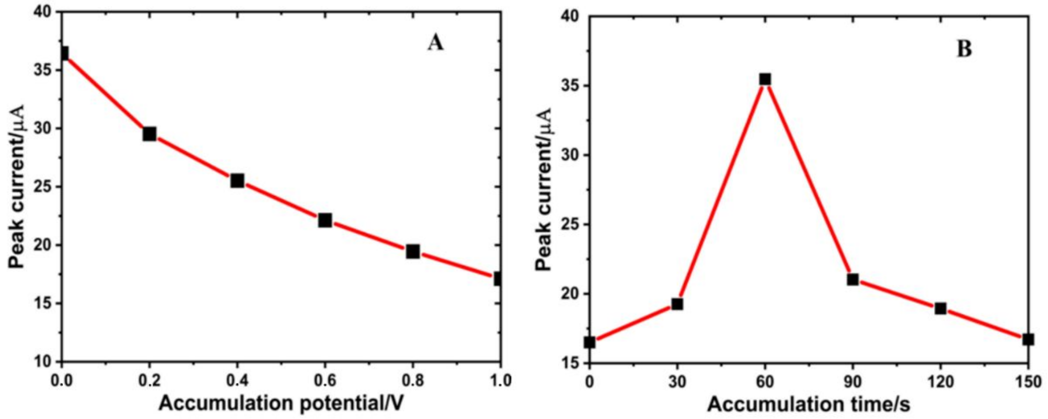

Figure S4. (A) Effect of the accumulation potential (0.0 V) and ((B) Effect of accumulation time (60s) on the peak current in 0.1 M H_2SO_4 using AdSDPV on MWCNTs/Fe_2O_3@Chitosan NC/GCE.
